# Supplementary material for: Deep learning predicts cardiovascular disease risks from lung cancer screening low dose computed tomography
Source: Nat Commun. 2021 May 20;12:2963. doi: 10.1038/s41467-021-23235-4 (PMC8137697; doi:10.1038/s41467-021-23235-4)
Supplement: Supplementary file 1 — Supplementary Information [file 41467_2021_23235_MOESM1_ESM.pdf]

Deep Learning Predicts Cardiovascular Disease Risks from Lung Cancer Screening Low Dose Computed Tomography

Hanqing Chao[1], Hongming Shan[1], Fatemeh Homayounieh[2], Ramandeep Singh[2],
Ruhani Doda Khera[2], Hengtao Guo[1], Timothy Su[3],
Ge Wang[1*], Mannudeep K. Kalra[2*], Pingkun Yan[1*]

[1]*Department of Biomedical Engineering, Biomedical Imaging Center, Rensselaer Polytechnic Institute, Troy NY 12180, USA*
[2]*Department of Radiology, Massachusetts General Hospital, Harvard Medical School, Boston MA 02114, USA*
[3]*Niskayuna High School, Niskayuna NY 12309, USA*
**Co-corresponding authors (wangg6@rpi.edu; mkalra@mgh.harvard.edu; yanp2@rpi.edu)*

Supplementary Information

Supplementary Notes

Model Structure Details. In feature extraction stage, three 2D CNN branches do not share parameters. The L+13 is employed on each slide separately, meaning each slide will have an extracted feature map. Then, a max pooling operation is employed along the dimension of slides to merge the feature maps of all slides into a single new feature map. Finally, the single feature map is feed to L-5 to form the representation of this view. Similarly, the attention blocks are also separately applied on each slide. After an attention map is generated, it is point-wisely multiplied with the features extracted by the L+13 to re-weight the feature. Following the conventional structure of attention in computer vision [1,2], the final feature feed to the subsequent net is the sum of the original feature and the reweighted feature.

Model Training and Test Details. The detected cardiac region is cut out from the original LDCT volume and resized to $128 \times 128 \times 128$ with Gaussian smoothing. All the loss functions in the model are the cross-entropy loss. The model was trained with the Adam optimizer [3]. The model without the attention blocks was first trained 10,000 iterations with batch size of 32 and learning rate of 1×10^{-4}. No learning rate decay strategy was applied. Checkpoints of the model were saved

every 100 iterations in the training stage. Next, the checkpoint achieved the highest performance on the NLST validation subset was selected to initialize the tuning of the whole model. The whole model was then tuned for another 1000 iterations with batch size of 16 and learning rate of 1×10^{-4} for the attention blocks and 1×10^{-5} for the rest. Checkpoints were also saved every 100 iterations and the best one on the NLST validation subset was selected as the final model. Data augmentations were used in all training, validation and test phases. In the training, an input $128 \times 128 \times 128$ 3D image was randomly cropped into $112 \times 112 \times 112$. In the validation and test phases, an input 3D image was augmented into 8 $112 \times 112 \times 112$ images with respect to 8 vertexes. The final classification probability is the average of 8 outputs. All models were trained, validated and tested on NVIDIA DGX-1 with 8 NVIDIA TESLA v100 GPUs. The codes were written in Python with PyTorch V1.4.0, Numpy V1.17.4, Apex V0.1 and TorchVision V0.5.0.

Glossary

- **CAC Score:** The coronary artery calcium (CAC) score is a semiqualitative measure of coronary calcification with ECG-gated, non-contrast CT. Agatston score [4] is used as a measure of CAC in this paper. It reflects the total area of calcium deposits and the density of the calcium in coronary artery.
- **CAD-RADS:** The Coronary Artery Disease - Reporting and Data System (CAD-RADS) [5] is an expert consensus document developed to standardize reporting of findings with coronary CT angiography.
- **MESA Score:** The Multi-Ethnic Study of Atherosclerosis (MESA) risk score [6] is an estimation of 10-year coronary heart disease risk obtained using traditional risk factors and coronary artery calcium.
- **CNN (a.k.a. deep CNN):** A convolutional neural network (CNN) is one class of deep neural networks that most commonly applied to visual images analysis.
- **DeepCAC:** A deep learning based system [7] designed for automatically calculating the CAC score from a chest CT image.
- **AE+SVM:** A two-stage machine learning based model [8] designed for CVD mortality prediction. It is composed by an auto-encoder (AE) for image feature extraction and a support vector machine (SVM) for classification.
- **KAMP-Net:** An end-to-end deep learning based model [9] designed for all-cause mortality prediction from a low-dose chest CT scan.
- **Grad-CAM:** Gradient-weighted Class Activation Mapping (Grad-CAM) [10] is a visualization approach for intuitive interpretation of decisions made by a convolutional neaural network based model. It uses the gradient of a target class flowing into the final convolutional layer to produce a coarse localization map highlighting important regions in an image for predicting the class.

Supplementary Figures

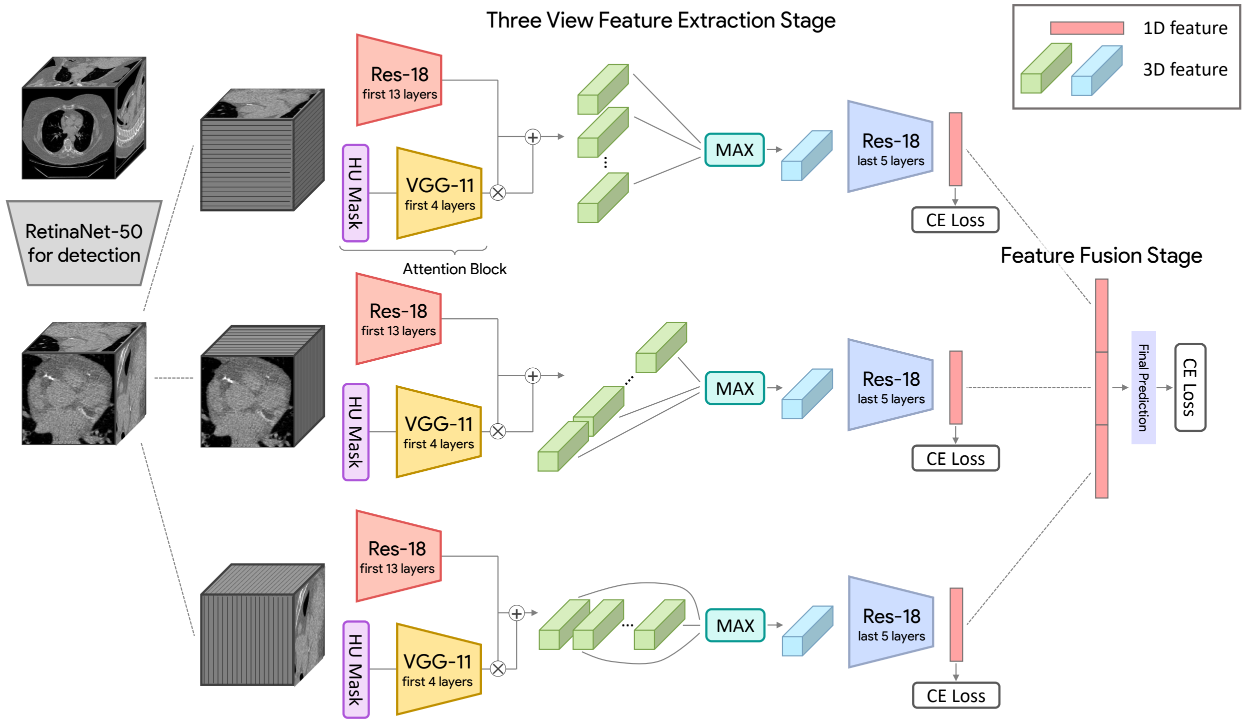

Supplementary Fig. 1: Framework of the proposed Tri2D-Net. The network contains two stages: the feature extraction stage and the feature fusion stage. The feature extraction stage consists of three 2D CNN branches to extract features from the three sequences of orthogonal views. The feature fusion stage aggregates the extracted features for classification.

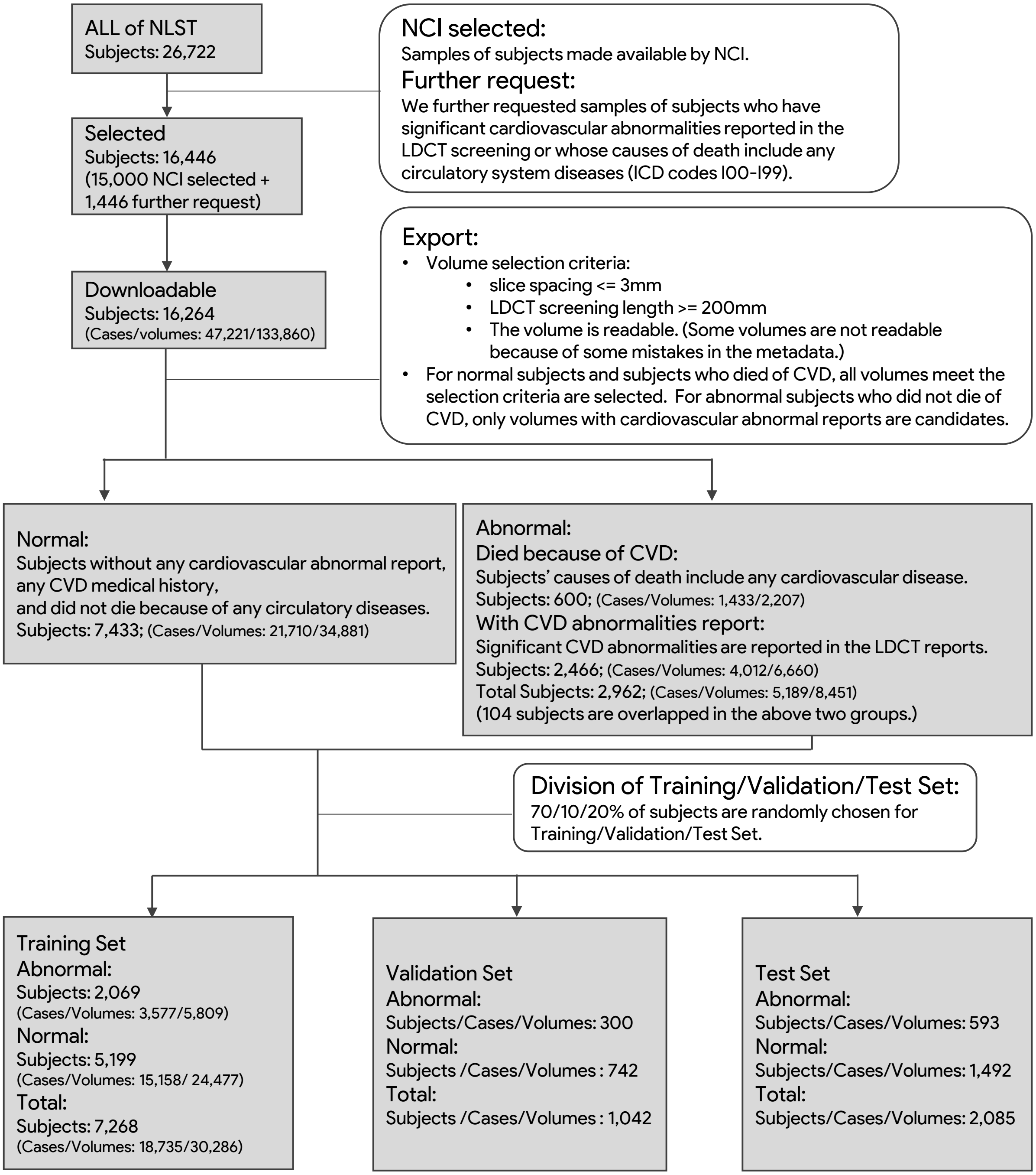

Supplementary Fig. 2: STARD flow diagram of the inclusion and exclusion of images in the NLST dataset used in our analysis.

Heart Disease Mortality Prediction

	AUC	95% Confidence Interval	p Value
Our Model	0.757	0.716 - 0.797	-
DeepCAC	0.721	0.676 - 0.766	0.0962
KAMP-Net	0.684	0.635 - 0.733	0.0025
AE+SVM	0.641	0.588 - 0.694	1e-5

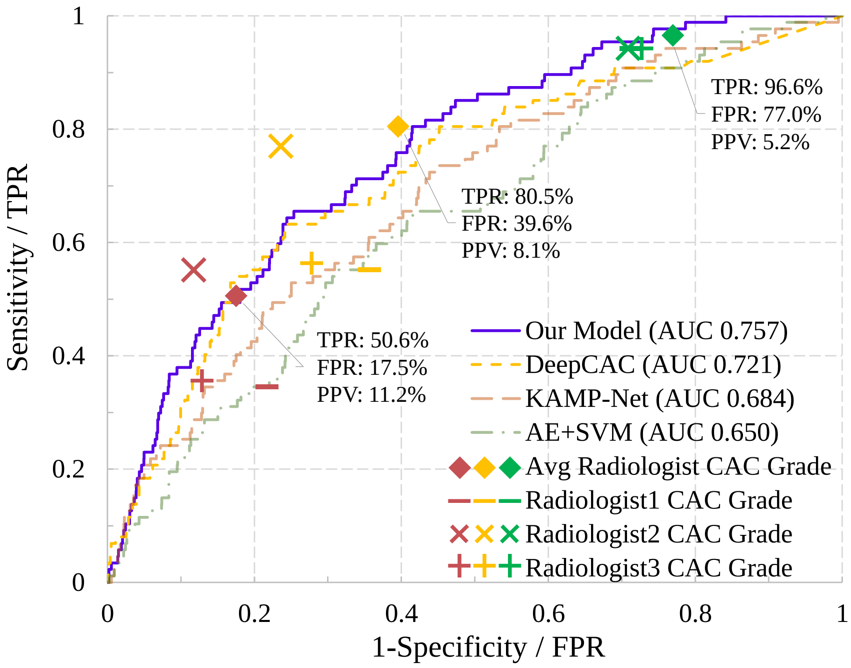

Supplementary Fig. 3: Experimental results on the NLST dataset for heart disease only mortality prediction. In stead of using all 17 ICD-10 codes in Supplementary Table 1, only heart disease related (I11, I20, I21, I24, and I25) deceases are regarded as positive cases (86 subjects). Our model exceeds other methods and achieved a performance similar to the average performance of human experts. p values were computed using a one-tail z-test.

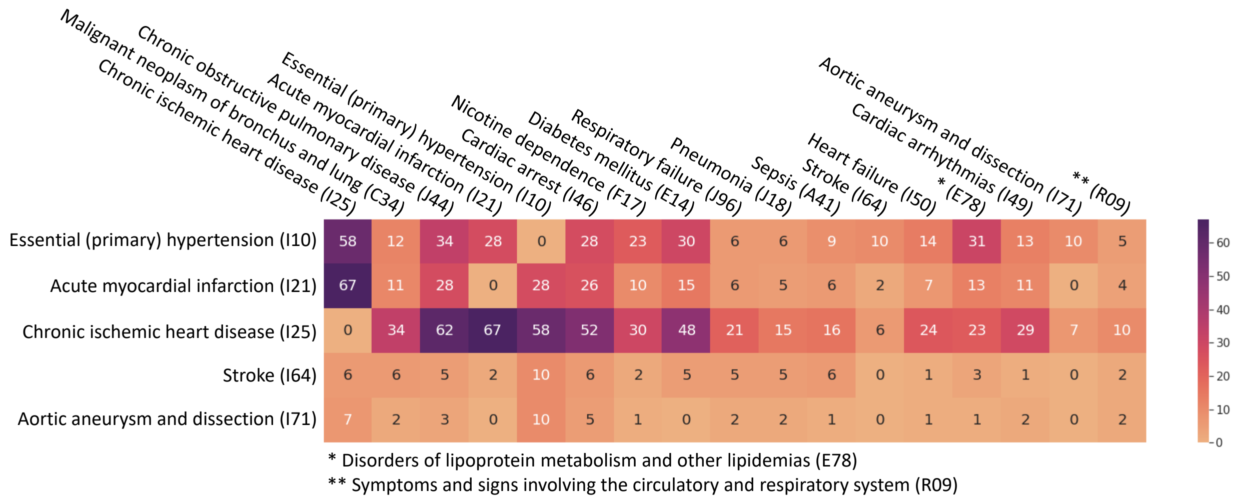

Supplementary Fig. 4: Causes of death co-occurance matirx. This matrix describes the co-occurance of 5 major CVD mortality causes (rows) and 17 overall major causes of death (columns) in NLST. For instance, the number 58 in the first row of the first column indicates that 58 deceased patients had both essential (primary) hypertension (I10) and chronic ischemic heart disease (I25) listed as their causes of death.

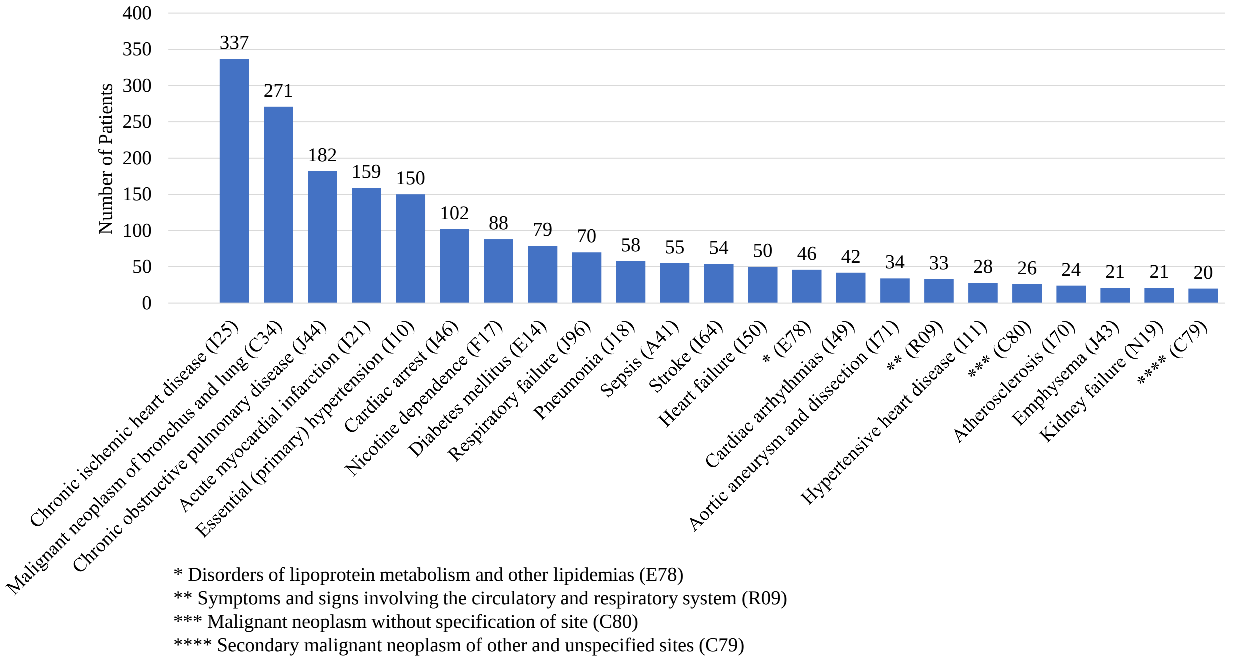

Supplementary Fig. 5: Histogram of major causes of death breakdown in the NLST dataset. Note that a patient might die from multiple causes.

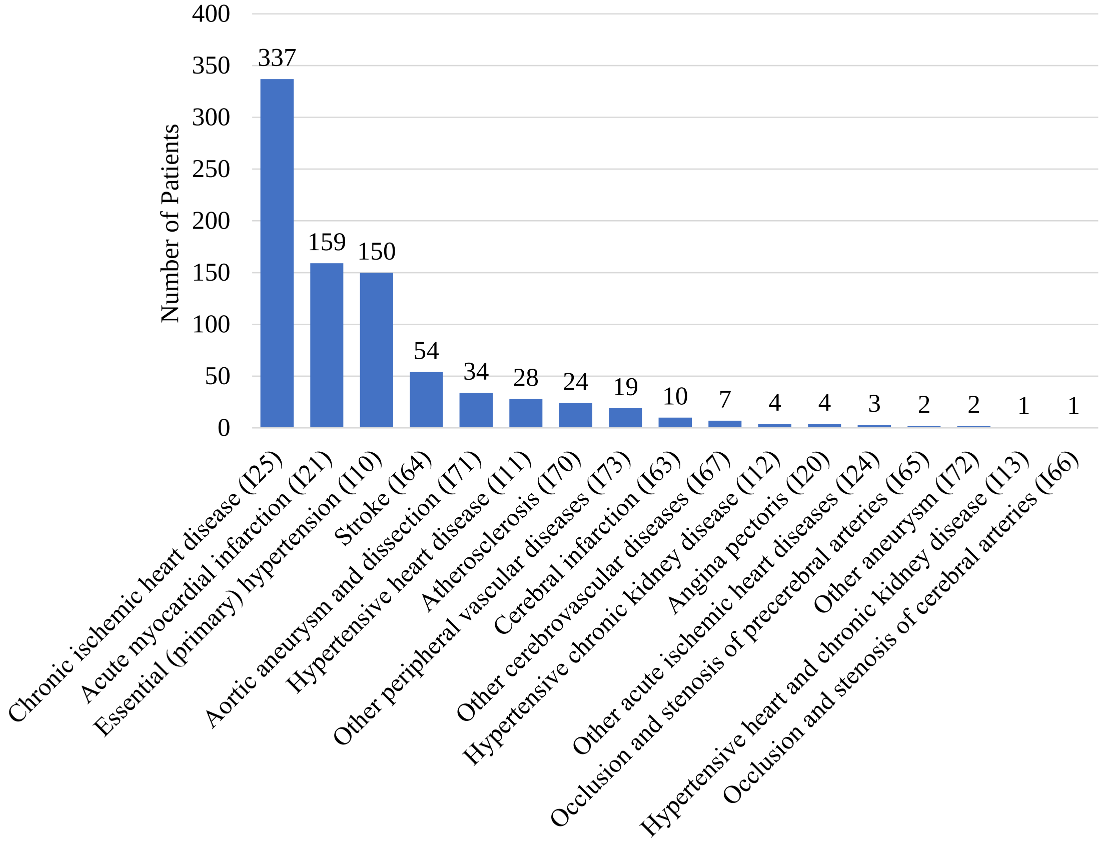

Supplementary Fig. 6: Histogram of the 17 CVD related causes of death in the NLST dataset. Note that a patient might die from multiple causes.

Supplementary Tables

Supplementary Table 1: Selected CVD-related causes of death.

Index	ICD-10 Code	Detail
1	I10	Essential (primary) hypertension
2	I11	Hypertensive heart disease
3	I12	Hypertensive renal disease
4	I13	Hypertensive heart and renal disease
5	I20	Angina pectoris
6	I21	Acute myocardial infarction
7	I24	Other acute ischemic heart diseases
8	I25	Chronic ischemic heart disease
9	I63	Cerebral infarction
10	I64	Stroke, not specified as haemorrhage or infarction
11	I65	Occlusion and stenosis of precerebral arteries
12	I66	Occlusion and stenosis of cerebral arteries
13	I67	Other cerebrovascular diseases
14	I70	Atherosclerosis
15	I71	Aortic aneurysm and dissection
16	I72	Other aneurysm
17	I73	Other peripheral vascular diseases

Supplementary Table 2: Manufacturers and scanner models used in the NLST dataset.

Manufacturer & Model	Volume Number	Reconstruction Kernels
GE MEDICAL SYSTEMS: CT scan	18	1. LUNG, 2 . BONE, 3 . STANDARD, 4 . BODY FILTER/STANDARD
GE MEDICAL SYSTEMS: Discovery LS	154	
GE MEDICAL SYSTEMS: Discovery QX/i	135	
GE MEDICAL SYSTEMS: HiSpeed QX/i	1596	
GE MEDICAL SYSTEMS: LightSpeed Plus	2378	
GE MEDICAL SYSTEMS: LightSpeed Power	14	
GE MEDICAL SYSTEMS: LightSpeed Pro 16	1529	
GE MEDICAL SYSTEMS: LightSpeed QX/i	4822	
GE MEDICAL SYSTEMS: LightSpeed Ultra	2491	
GE MEDICAL SYSTEMS: LightSpeed VCT	6	
GE MEDICAL SYSTEMS: LightSpeed16	3872	
GE MEDICAL SYSTEMS: QX/i	3	
Philips: Mx8000	2382	1. D, 2. C, 3. B
Philips: Mx8000 IDT	90	
Philips: Mx8000 IDT 16	65	
SIEMENS: Emotion 16	14	1. B50f, 2. B45f, 3. B50s, 4. B60f, 5. B60s, 6. B70f, 7. B30f, 8. B31s, 9. B80f, 10. B20f, 11. B30s, 12. B31f
SIEMENS: Emotion 6	3	
SIEMENS: Sensation 10	2	
SIEMENS: Sensation 16	3870	
SIEMENS: Sensation 4	706	
SIEMENS: Sensation 64	393	
SIEMENS: Volume Zoom	7001	
TOSHIBA: Aquilion	1869	1. FC51, 2. FC50, 3. FC53, 4. FC30, 5. FC10, 6. FC82, 7. FC02, 8. FC01
**Summary**	**33,413**	

Supplementary Table 3: Manufacturers and scanner models used in the MGH dataset.

Manufacturer & Model	Volume Number	Reconstruction Kernels
LDCT		
GE MEDICAL SYSTEMS: Discovery CT750 HD	68	1. STANDARD
GE MEDICAL SYSTEMS: Discovery STE	13	
GE MEDICAL SYSTEMS: LightSpeed Pro 16	18	
GE MEDICAL SYSTEMS: LightSpeed VCT	29	
GE MEDICAL SYSTEMS: Revolution CT	51	
Philips : Brilliance 40	5	1. B
Philips : iCT 256	42	
Philips : IQon - Spectral CT	57	
SIEMENS : Biograph 64	3	1. I30f\3, 2. I30f\2, 3. Br40d\3, 4. I31f\3, 5. B30f, 6. Br40f\2
SIEMENS : SOMATOM Definition Edge	35	
SIEMENS : SOMATOM Definition Flash	4	
SIEMENS : SOMATOM Drive	2	
SIEMENS : SOMATOM Force	8	
**Summary**	**335**	
ECG-gated CCT		
SIEMENS : SOMATOM Force	84	1. B35f, 2. B31f
SIEMENS : SOMATOM Definition Flash	151	
**Summary**	**235**	

References

1. Wang, X., Girshick, R., Gupta, A. & He, K. Non-local neural networks. In *Proceedings of the IEEE conference on computer vision and pattern recognition*, 7794–7803 (2018).

2. Fu, J. *et al.* Dual attention network for scene segmentation. In *Proceedings of the IEEE/CVF Conference on Computer Vision and Pattern Recognition*, 3146–3154 (2019).

3. Kingma, D. P. & Ba, J. Adam: A method for stochastic optimization. *arXiv preprint arXiv:1412.6980* (2014).

4. Agatston, A. S. *et al.* Quantification of coronary artery calcium using ultrafast computed tomography. *Journal of the American College of Cardiology* **15**, 827–832 (1990).

5. Cury, R. C. *et al.* CAD-RADS^{TM} coronary artery disease–reporting and data system. an expert consensus document of the Society of Cardiovascular Computed Tomography (SCCT), the American College of Radiology (ACR) and the North American Society for Cardiovascular Imaging (NASCI). Endorsed by the American College of Cardiology. *Journal of cardiovascular computed tomography* **10**, 269–281 (2016).

6. McClelland, R. L. *et al.* 10-year coronary heart disease risk prediction using coronary artery calcium and traditional risk factors. *Journal of the American College of Cardiology* **66**, 1643–1653 (2015).

7. Zeleznik, R. *et al.* Deep convolutional neural networks to predict cardiovascular risk from computed tomography. *Nature Communications* **12**, 1–9 (2021).

8. van Velzen, S. G. *et al.* Direct prediction of cardiovascular mortality from low-dose chest ct using deep learning. In *Medical Imaging 2019: Image Processing*, vol. 10949, 109490X (International Society for Optics and Photonics, 2019).

9. Guo, H., Kruger, U., Wang, G., Kalra, M. K. & Yan, P. Knowledge-based analysis for mortality prediction from CT images. *IEEE Journal of Biomedical and Health Informatics* **24**, 457–464 (2020).

10. Selvaraju, R. R. *et al.* Grad-cam: Visual explanations from deep networks via gradient-based localization. In *Proceedings of the IEEE international conference on computer vision*, 618–626 (2017).
