# Supplementary material for: Deep learning predicts cardiovascular disease risks from lung cancer screening low dose computed tomography
Source: Nat Commun. 2021 May 20;12:2963. doi: 10.1038/s41467-021-23235-4 (PMC8137697; doi:10.1038/s41467-021-23235-4)
Supplement: Supplementary file 3 — Reporting Summary [file 41467_2021_23235_MOESM3_ESM.pdf]

nature research

Corresponding author(s): Pingkun Yan
Mannudeep K. Kalra
Ge Wang

Last updated by author(s): Apr 15, 2021

# Reporting Summary

Nature Research wishes to improve the reproducibility of the work that we publish. This form provides structure for consistency and transparency in reporting. For further information on Nature Research policies, see our Editorial Policies and the Editorial Policy Checklist.

## Statistics

For all statistical analyses, confirm that the following items are present in the figure legend, table legend, main text, or Methods section.

| n/a | Confirmed | |
|---|---|---|
| ☐ | ☒ | The exact sample size ($n$) for each experimental group/condition, given as a discrete number and unit of measurement |
| ☐ | ☒ | A statement on whether measurements were taken from distinct samples or whether the same sample was measured repeatedly |
| ☐ | ☒ | The statistical test(s) used AND whether they are one- or two-sided<br>*Only common tests should be described solely by name; describe more complex techniques in the Methods section.* |
| ☐ | ☒ | A description of all covariates tested |
| ☐ | ☒ | A description of any assumptions or corrections, such as tests of normality and adjustment for multiple comparisons |
| ☐ | ☒ | A full description of the statistical parameters including central tendency (e.g. means) or other basic estimates (e.g. regression coefficient) AND variation (e.g. standard deviation) or associated estimates of uncertainty (e.g. confidence intervals) |
| ☐ | ☒ | For null hypothesis testing, the test statistic (e.g. $F$, $t$, $r$) with confidence intervals, effect sizes, degrees of freedom and $P$ value noted<br>*Give P values as exact values whenever suitable.* |
| ☒ | ☐ | For Bayesian analysis, information on the choice of priors and Markov chain Monte Carlo settings |
| ☐ | ☒ | For hierarchical and complex designs, identification of the appropriate level for tests and full reporting of outcomes |
| ☒ | ☐ | Estimates of effect sizes (e.g. Cohen's $d$, Pearson's $r$), indicating how they were calculated |

*Our web collection on statistics for biologists contains articles on many of the points above.*

## Software and code

Policy information about availability of computer code

| | |
|---|---|
| Data collection | Dicom files were handled with the free dicom viewer MicroDicom DICOM Viewer V3.01 (https://www.microdicom.com/) |
| Data analysis | Data analysis was conducted in Python 3.7. The major dependent packages are Apex V0.1, Numpy V1.17.4, PyTorch V1.4docke.0 and TorchVision V0.5.0. The code of the proposed method is available on Github (https://github.com/DIAL-RPI/CVD-Risk-Estimator) |

For manuscripts utilizing custom algorithms or software that are central to the research but not yet described in published literature, software must be made available to editors and reviewers. We strongly encourage code deposition in a community repository (e.g. GitHub). See the Nature Research guidelines for submitting code & software for further information.

## Data

Policy information about availability of data

All manuscripts must include a data availability statement. This statement should provide the following information, where applicable:

- Accession codes, unique identifiers, or web links for publicly available datasets
- A list of figures that have associated raw data
- A description of any restrictions on data availability

The NLST dataset used in this study is publicly available from the NIH. Details can be found at https://biometry.nci.nih.gov/cdas/learn/nlst/images/
The MGH dataset was not part of the NLST dataset. The de-identified dataset from MGH was used with an institutional review board-approval for the current study. The imaging data from MGH cannot be shared publicly due to patient privacy concerns.

# Field-specific reporting

Please select the one below that is the best fit for your research. If you are not sure, read the appropriate sections before making your selection.

- [x] Life sciences
- [ ] Behavioural & social sciences
- [ ] Ecological, evolutionary & environmental sciences

For a reference copy of the document with all sections, see nature.com/documents/nr-reporting-summary-flat.pdf

# Life sciences study design

All studies must disclose on these points even when the disclosure is negative.

**Sample size**

The National Lung Screening Trial (NLST) enrolled 26,722 participants to undergo three annual screening exams with LDCT. We used 33,413 CT volumes from 10,395 subjects. They were randomly divided into training (7,268, 70%), validation (1,042, 10%) and test sets (2,085, 20%). This scheme follows a standard way of splitting datasets for deep learning research. We believe that this sample size is sufficient as it represents all 33 sites in the NLST trial with CT exams from all major CT manufacturers.

The independent test dataset collected at Massachusetts General Hospital (MGH at Boston, MA) contains 335 patients. By reviewing the electronic medical records (EPIC, Epic Systems Corporation) at MGH, we first identified all available 235 subjects who had clinically indicated LDCT for lung cancer screening, ECG-gated CT angiography and coronary calcium scoring within a 12-month period. Further, to expand the population, we randomly collected the other 100 subjects with clinically indicated LDCT for lung cancer screening. For the one tail z-test conducted to compare different models, a sample size of 335 gives a test power of 0.9999993.

The reader studies assessed all chest LDCTs within the test set on NLST datasets (2,085 patients).

We trained and validated our deep learning model on a part of NLST dataset (8,310 patients). The remaining NLST and the entire MGH datasets were held back from the development of our deep learning model for the testing phase of our study.

**Data exclusions**

NLST dataset:
We received 133,860 CT volumes of 16,264 subjects from NCI, which has reached the maximal number of cases allowed for a public study. Subjects were excluded when they can neither be determined as CVD-positive (who have no reported cardiovascular abnormality in any of the CT scans during the trial, and did not die of circulatory system diseases) nor CVD-negative (who have CVD related medical history). CT volumes were excluded with slice spacing larger than 3mm, or screening length smaller than 200mm, or that are not readable. For abnormal subjects who did not die of CVD, CT volumes without cardiovascular abnormal reports were excluded. This exclusion process resulted in 34,881 CT volumes from CVD-negative subjects and 8,451 CT volumes from CVD-positive subjects. Finally, for each of the 3,127 subjects in the validation and test sets, only the volume with the earliest timestamp was included for keeping the real data distribution. All the exclusion criteria were pre-established.

MGH dataset:
Images and information of 348 subjects were collected at MGH. Thirteen subjects were excluded because they had coronary stents, prostheticheart valves, prior coronary artery bypass graft surgery, or metal artifacts in the region of cardiac silhouette. The exclusion criteria were pre-established.

**Replication**

All attempts at replication were successful. Our findings persisted through numerous retrainings with random network initialization and training data iteration order. The high performance of our model was replicated on the completely independent MGH dataset. In the reader studies, three radiologists evaluated the test images independently in a double-blinded fashion. The code of our model and the trained parameters to replicate the statistical analysis are shared.

**Randomization**

For NLST, patients were randomly assigned into the training, validation or test sets. All CT volumes and meta data from each patient were associated with the same split as the patient.

No randomization was performed on the MGH dataset. The whole dataset was only used for the testing phase of our study.

**Blinding**

We trained and validated our deep learning model on a part of NLST dataset (the training and validation sets). The remaining NLST and the entire MGH datasets were held back from the development of our deep learning model for the testing phase of our study.
In reader studies, none of the radiologists who interpreted the images (either in the course of clinical practice or in the context of the reader study) had knowledge of any aspect of the deep learning model.
In the collection of the MGH dataset, we were blinded to the result of chest LDCT screening, ECG-gated CT angiography and coronary calcium scoring.

# Reporting for specific materials, systems and methods

We require information from authors about some types of materials, experimental systems and methods used in many studies. Here, indicate whether each material, system or method listed is relevant to your study. If you are not sure if a list item applies to your research, read the appropriate section before selecting a response.

## Materials & experimental systems

| n/a | Involved in the study | |
|---|---|---|
| ☒ | ☐ | Antibodies |
| ☒ | ☐ | Eukaryotic cell lines |
| ☒ | ☐ | Palaeontology and archaeology |
| ☒ | ☐ | Animals and other organisms |
| ☐ | ☒ | Human research participants |
| ☒ | ☐ | Clinical data |
| ☒ | ☐ | Dual use research of concern |

## Methods

| n/a | Involved in the study | |
|---|---|---|
| ☒ | ☐ | ChIP-seq |
| ☒ | ☐ | Flow cytometry |
| ☒ | ☐ | MRI-based neuroimaging |

# Human research participants

Policy information about studies involving human research participants

| | |
|---|---|
| Population characteristics | NLST dataset: We included 6,115 man and 4,280 women with mean age +/- standard deviation of 61 +/- 5 years (age range 54-74 years).<br><br>MGH dataset: The 335 subjects are composed by 161 males and 174 females with mean age +/- standard deviation of 64 +/- 8 years. |
| Recruitment | De-identified data from NLST were acquired and accessed with approvals from both the RPI and the NIH data source. Retrospective use of MGH dataset was approved by the human research committee of the IRB with the waiver of informed consent. The study was in compliance with the Health Insurance Portability and Accountability Act (HIPAA). |
| Ethics oversight | All participants enrolling in NLST signed an informed consent developed and approved by the IRBs at each of the 33 participating medical institutions, the National Cancer Institute (NCI) IRB, and the Westat IRB. Further details of the NLST are available through the Cancer Data Access System (CDAS) of the National Institutes of Health (NIH).<br>The Human Research Committee of the MGH IRB approved this HIPAA compliant prospective clinical study. Also, the Human Research Committee of the RPI IRB approved the use of these patient data. |

Note that full information on the approval of the study protocol must also be provided in the manuscript.
